# Sex-Specific Prediction Models of Alzheimer's Disease: A Gene Expression Analysis

**DOI:** 10.7150/ijms.122666

**Published:** 2026-01-30

**Authors:** Xiaomeng Ma, Abdilahi Abdi Ibrahim, Lili Ma, Xueying Ma, Zhan Ma, Yingying Liu, Donghong Li, Jia Liu, Xiaofeng Xu, Huimin Dong, Xiaohong Chen, Fuhua Peng

**Affiliations:** 1 Department of Neurology, The Third Affiliated Hospital of Sun Yat-Sen University, Guangzhou, China.; 2 Department of Laboratory Medicine, The Third Affiliated Hospital of Sun Yat-sen University, Guangzhou, China.

**Keywords:** Alzheimer's disease, bioinformatics, gene expression, sex-specific

## Abstract

Alzheimer's disease (AD) exhibits sex-specific molecular signatures that may improve diagnostic precision. We aimed to identify and validate male- and female-specific blood and brain gene expression biomarkers for AD prediction. We analyzed four GEO datasets (blood- and brain-derived) using limma and Fisher's meta-analysis to identify sex-specific differentially expressed genes, assessed age associations via linear regression, and constructed 10-fold cross-validated logistic regression models. After performing a meta-analysis, 74 differentially expressed genes were identified in the female cohort and 89 DEGs were screened in the male cohort. ERH and MRPS33 were identified as the most relevant genes in the male cohort, and NDUFA1 and NDUFS5 were screened in the female cohort. The identified genes were downregulated in AD samples compared to controls. Both male-specific and female-specific prediction models achieved an AUC of above 0.7 in two external validation blood-derived datasets as well entorhinal cortex dataset. Paradoxically, qPCR showed significant upregulation of all four genes in the AD group compared to the control group.

## Introduction

Alzheimer's disease (AD) represents a major global health concern, impacting millions of individuals worldwide, and is characterized by progressive cognitive decline and memory impairment 1. Genetics of AD has been extensively studied since the end of the previous century. One of the rare forms of AD, known as early-onset AD, is primarily caused by mutations in the APP, PSEN1, and PSEN2 genes, which alter the processing of amyloid precursor protein. In turn, this increases the production of Aβ42, a core event in the amyloid cascade hypothesis 2-4. For the much more common late-onset AD, the ε4 allele of the APOE gene is the only established genetic risk factor associated with the development of AD 5. Apart from these core genes, genome-wide association studies have revealed numerous susceptibility genes, such as CLU, BIN1, and TREM2, implicating additional biological pathways involved in pathogenesis of AD (excellently summarized in a recent review 6).

Emerging evidence suggests that biological sex plays a critical role in AD pathophysiology, influencing disease risk, progression, and response to treatment 7, 8. Understanding sex-specific differences in AD has garnered increasing attention in neurology research, highlighting the need for tailored approaches 9. Women were reported to have a higher prevalence of AD and have different clinical presentations compared to men 10. Sex-specific differences extend beyond prevalence and clinical presentation and include genetic, hormonal, and immunological factors 11, 12. Several studies have emphasized sex-specific differences in gene expression profiles, implicating unique molecular pathways underlying disease pathogenesis. While the APOE ε4 allele presents risk in both sexes, women carriers show a higher incidence of AD between 65 and 75 years and exhibit faster cognitive decline compared to male carriers 13. Interestingly, higher prevalence of AD in women is observed despite the fact that overall measures of Aβ burden often do not show consistent sex differences 14. Recent studies investigated association between AD and genes previously not implicated with its progression. It was recently discovered that LRP10 acts as a major regulator in the female AD network and may be crucial in driving sex-specific differences in AD development 11. Another study revealed that increased expression of SERPINB1, SERPINB6 and SERPINB9 in the prefrontal cortex of female AD patients was associated with significantly higher levels of amyloidosis. In contrast, such differences were not observed in male AD patients 15.

Despite advances in our understanding of AD, there is still a lack of studies covering sex-specific differences in terms of gene expression of patients diagnosed with AD. Our study was conducted in order to contribute to the exploration of sex-specific gene expression profiles with the primary objective of creating sex-specific prediction models capable of predicting AD based on gene expression profiles alone.

## Methods

### Datasets

The study was conducted with four datasets obtained from the Gene Expression Omnibus that included patients with AD and controls: GSE140829, GSE63060, GSE63061, and GSE118553. All datasets provided information on age and gender. GSE140829, GSE63060 and GSE63061 contained blood samples of patients diagnosed with AD, mild cognitive impairment (MCI), and controls. GSE118553 included brain tissue samples obtained from various regions (temporal cortex, frontal cortex, entorhinal cortex, and cerebellum) of AD, asymptomatic AD, and control subjects.

#### GSE140829

A total of 587 samples comprised the GSE140829 dataset, including 204 AD, 249 control, and 134 MCI subjects. Following the removal of MCI samples, the dataset was divided into two based on gender. The male-only subset consisted of 100 AD samples and 110 non-AD samples, whereas the female-only subset included 104 AD samples and 139 controls. GSE140829 was used as a discovery set. Male and female subsets were normalized and adjusted for batch effects using limma and sva packages, respectively. Each dataset was preprocessed using 'neqc' function available in limma (normexp background correction and quantile normalization).

#### GSE63060

In GSE63060, three samples (4856050008_B, 4856050008_K, and 4856050048_A) were removed from expression data as they were not found in phenodata, and one sample (4856076038_D) was removed from phenodata as it was not found in expression data. Then, 80 samples with MCI were removed, leaving 142 AD and 104 control samples. Among 246 samples, there were 158 females (96 with AD and 62 controls) and 88 males (46 with AD and 42 controls). Normexp background correction and quantile normalization were applied to each dataset.

GSE63060 was used as the first external validation set.

#### GSE63061

The GSE63061 dataset contained 388 samples: 139 AD, 109 MCI, 134 controls, three borderline MCI, and three unspecific samples. All samples except for AD and controls were removed. The final dataset included 273 samples: 166 females (85 with AD and 81 controls) and 107 males (54 with AD and 53 controls). Normexp background correction and quantile normalization were applied to each dataset. GSE63061 was used as the second external validation set.

#### GSE118553

In the GSE118553, 115 samples were obtained from the temporal cortex: 51 males (19 controls, 23 AD, and 9 asymptomatic AD samples) and 64 females (12 controls, 29 AD, and 23 asymptomatic AD samples). There were 92 samples in the cerebellum: 41 males (12 controls, 18 AD, and 11 asymptomatic AD samples) and 51 females (10 controls, 20 AD, and 21 asymptomatic AD samples). In the entorhinal cortex, there were 98 samples: 36 males (12 controls, 14 AD, and 10 asymptomatic AD samples) and 62 females (12 controls, 23 AD, and 27 asymptomatic AD samples). Finally, frontal cortex consisted of 96 samples: 36 males (12 controls, 14 AD, and 10 asymptomatic AD samples) and 60 females (11 controls, 26 AD, and 23 asymptomatic AD samples). Thus, the GSE118553 dataset was split into four separate datasets based on the tissue origin, and each one was used to validate the predictive models. Each dataset was preprocessed using 'neqc' function available in limma (normexp background correction and quantile normalization).

### Identification of Differentially Expressed Genes

For each blood-derived dataset (GSE140829, GSE63060, GSE63061), differentially expressed genes (DEGs) between AD and controls were identified separately for males and females using limma (empirical Bayes statistics). A meta-analysis (Fisher's) was then conducted for sex-specific datasets using ExpressAnalyst with the following cut-off values: p-value < 0.05 and |logFC| > mean(|logFC|) + 2*SD*(|logFC|), which was equal to 0.32 for females and 0.37 for males. Kyoto Encyclopedia of Genes and Genomes (KEGG) analysis was used to identify enriched pathways.

### Development and Validation of Predictive Models

A univariate feature selection was applied to identify the most relevant genes that could result in the best performance. Feature selection is essential to reduce the dimensionality of the training set as a model cannot be trained on all genes found in the dataset. Feature selection reduces the computational power required to do the analysis, improves the generalization ability and interpretability of machine learning models, enhances prediction accuracy, and reduces the risk of model overfitting. In order to identify AD based on gene expression, a logistic regression machine learning algorithm (available within caret package) was used with 10-fold cross validation. Using the identified genes, models were trained on GSE140829 and validated on GSE63060 and GSE63061. In addition, GSE118553 was used as separate validation set to explore the predictive performance of models in brain tissues. Sensitivity, specificity, and area under the curve (AUC) were calculated to assess model's performance.

### Experimental validation

The whole venous blood was collected prospectively from patients with confirmed Alzheimer's disease and controls without any signs of dementia starting from May 1, 2024 to April 1, 2025 at the Third Affiliated Hospital of Sun Yat-Sen University.

For blood samples, a volume of 500 μL of whole blood was transferred into a 15 mL centrifuge tube, to which ten volumes of red blood cell lysis buffer were added. The mixture was gently inverted and incubated at room temperature, protected from light, for 10 minutes. The sample was then centrifuged at 300 g for 10 minutes at room temperature, and the supernatant was discarded. This red blood cell lysis procedure was repeated once under the same conditions. The resulting cell pellet was resuspended in PBS and centrifuged again at 300 g for 10 minutes at room temperature, after which the supernatant was removed. The remaining cells were resuspended in 100 μL PBS, followed by the addition of 1 mL TRIpure reagent. After thorough mixing, the sample was left to stand for 5 minutes. Next, 250 μL of chloroform was added, mixed thoroughly, and incubated on ice for 5 minutes. The mixture was then centrifuged at 10,000 g for 10 minutes at 4 °C. In a biosafety cabinet, 500 μL of the upper aqueous phase was carefully transferred to a 1.5 mL EP tube, mixed with an equal volume of ice-cold isopropanol, and incubated at -20 °C for 15 minutes. After another centrifugation at 10,000 g for 10 minutes at 4 °C, the supernatant was carefully removed, and the RNA pellet was washed with 1 mL of 75% ethanol pre-chilled at 4 °C. The tube was inverted several times to ensure proper washing, then centrifuged at 10,000 g for 5 minutes at 4 °C. The supernatant was discarded, and the pellet was allowed to air-dry for several minutes in a biosafety cabinet. Finally, the RNA pellet was dissolved in 100 μL of RNase-free water.

First-strand cDNA synthesis was performed using the EntiLink™ 1st Strand cDNA Synthesis Kit (ELK Biotechnology, EQ003). On ice, a reaction mixture was prepared containing 2.0 μL of RT primer mix, 1.0 μL of 2.5 mM dNTPs, and a volume of RNA adjusted to reach a final volume of 15.0 μL. This mixture was incubated at 70 °C for 5 minutes in a thermal cycler and immediately chilled on ice for 2 minutes. Subsequently, the second step reaction mix was prepared on ice by adding 4.0 μL of 5× RT buffer, 1.0 μL of M-MLV reverse transcriptase, and 1.0 μL of RNase inhibitor to the previous mixture. The complete reaction was incubated at 37 °C for 60 minutes, followed by enzyme inactivation at 85 °C for 5 minutes, and then held at 4 °C.

Quantitative real-time PCR (qPCR) was carried out on the StepOne™ Real-Time PCR System (Life Technologies), using the EnTurbo™ SYBR Green PCR SuperMix Kit (ELK Biotechnology, EQ001). Each sample was run in triplicate. The thermal cycling protocol included an initial denaturation step at 95 °C for 3 minutes, followed by 40 cycles of denaturation at 95 °C for 10 seconds, annealing at 58 °C for 30 seconds, and extension at 72 °C for 30 seconds. The melting curve analysis was performed using the instrument's default settings. The total reaction volume was 10 μL, composed of 5.0 μL of 2× Master Mix, 1.0 μL of 2.5 μM primer working solution, 1.0 μL of template cDNA, 2.0 μL of double-distilled water, and 1.0 μL of ROX reference dye. List of primers is shown in Table 1.

### Statistical Analysis

Homogeneity of variance was assessed using Levene's test, and normality was assessed using Shapiro-Wilk's test. A student's t-test was used to calculate statistical significance between AD and control samples if equal variance and normality were assumed. When normal distribution was not assumed, Mann-Whitney U test was performed. P-value < 0.05 was considered statistically significant. Pearson correlation coefficients were computed for expression levels of the identified genes. The correlation was visualized using scatter plots with linear regression lines, and corresponding correlation coefficients (r) and p-values were calculated. In addition, the association between age and gene expression levels was conducted. For each group, linear regression models were fitted and the relationship was visualized using scatter plots with regression lines. The p-values for the association between age and expression were computed separately for each group.

Data analysis of qPCR results was conducted using the ΔΔCT method. For each sample, ΔCT was calculated as the difference between the CT value of the target gene and that of the internal reference gene (GAPDH). The ΔΔCT value was obtained by subtracting the ΔCT of the control sample from that of the experimental sample. Fold change in gene expression was then calculated as 2^-ΔΔCT^. Statistical analysis was performed in R using cor package. All graphics, including volcano plot, violin plot and others, were performed using ggplot2 package.

## Results

### Identification of Differentially Expressed Genes

**Figure 1:**
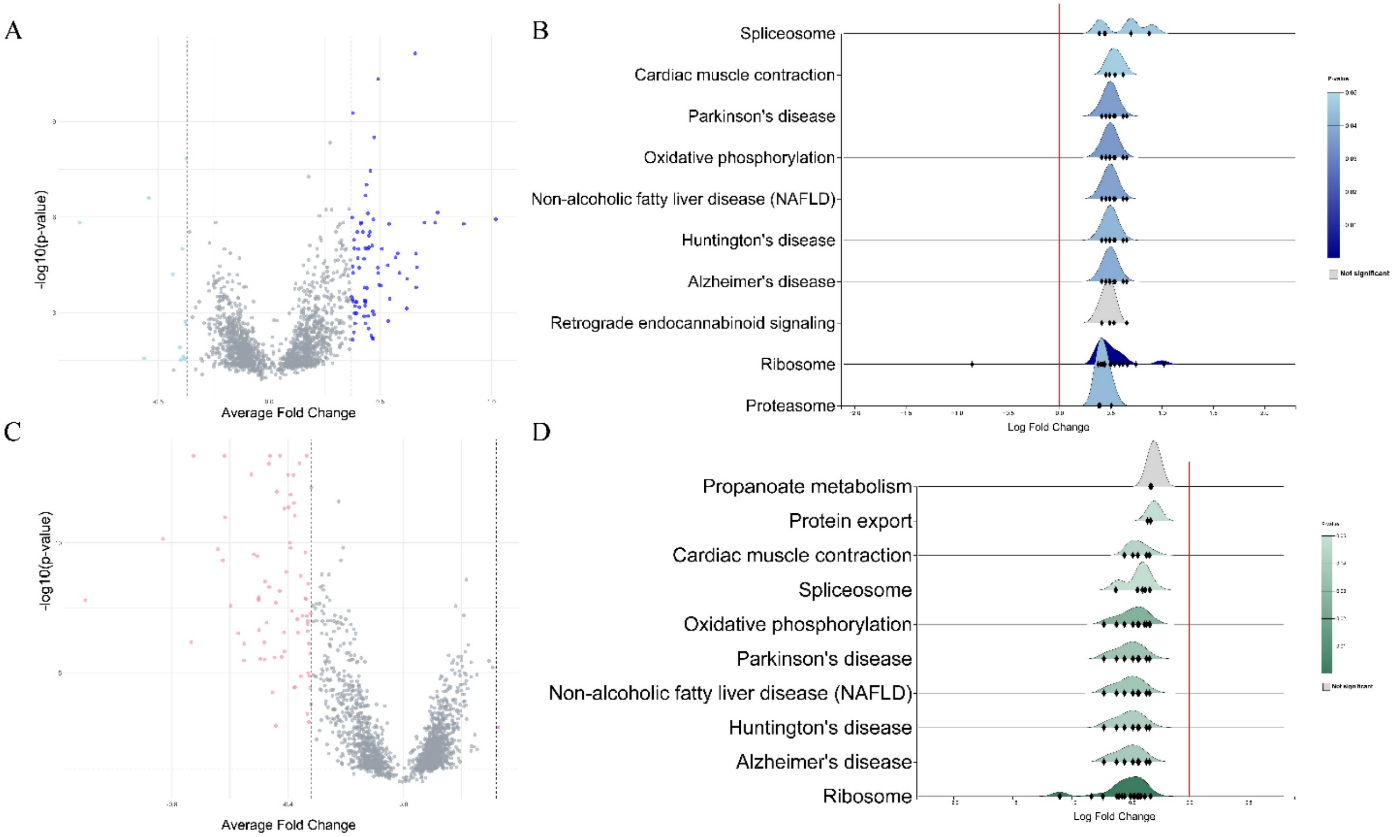
**A.** Volcano plot of differentially expressed genes in males. **B.** Rigline plot of fold change distribution (males). **C.** Volcano plot of differentially expressed genes in females. **D.** Rigline plot of fold change distribution (females).

Each dataset was split into male and female sets and then normalized. Datasets that analyzed gene expression in blood specimens were used for differential expression analysis and subsequent meta-analysis of DEGs. In females, a total of 700 DEGs were extracted from the GSE140829 dataset, 1809 from the GSE63060 dataset, and 457 from the GSE63061 dataset. In males, 1099 DEGs were identified in the GSE63061, 1703 in the GSE63060, and 320 in the GSE140829 datasets. After performing a meta-analysis, 74 DEGs were identified in the female cohort and 89 DEGs were screened in the male cohort (Figure 1). In both males and females, KEGG enrichment analysis revealed genes were mainly enriched in similar pathways, such as ribosome, Alzheimer's disease, non-alcoholic fatty liver disease, etc.

### Gene Selection and Analysis

**Figure 2:**
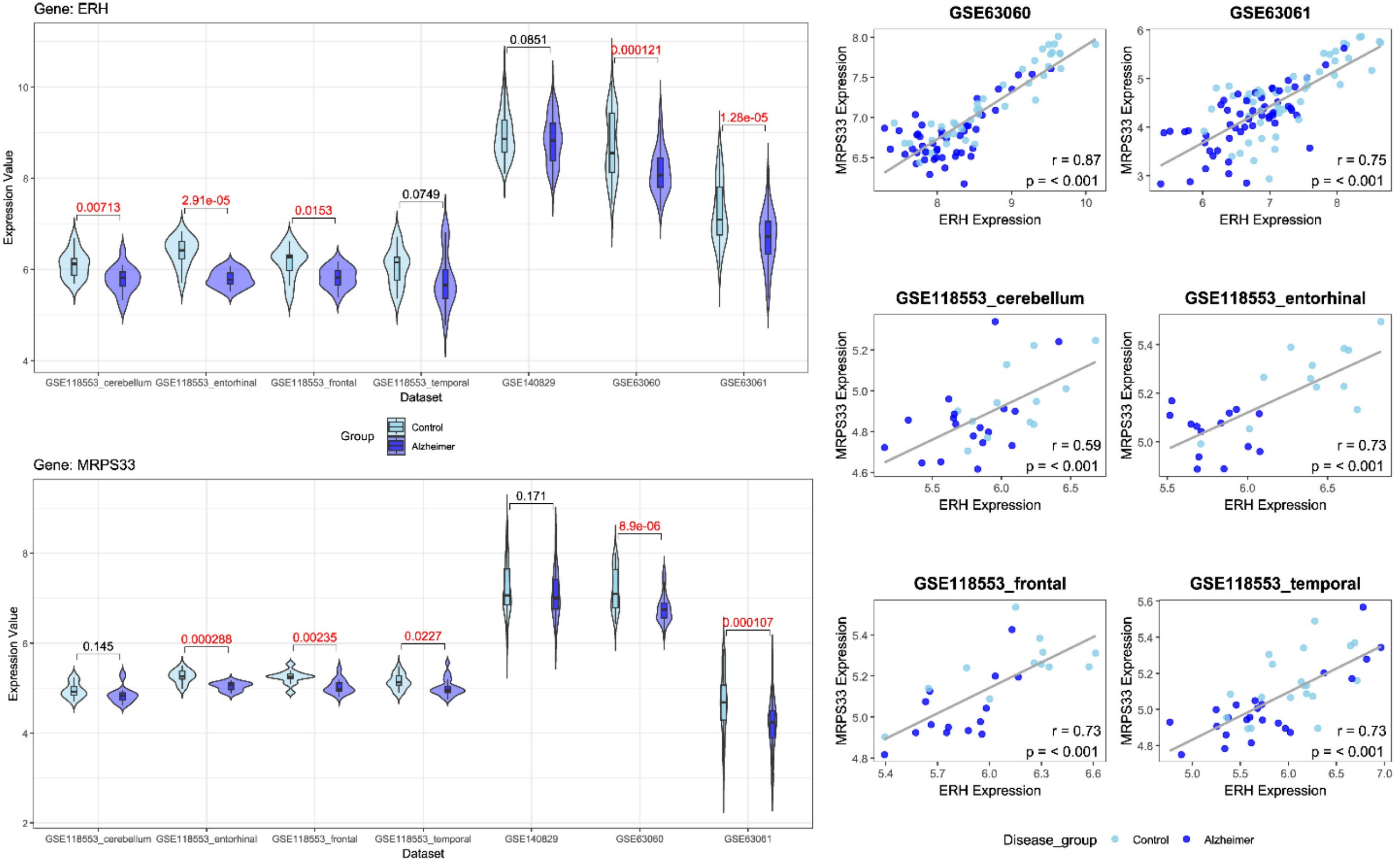
**Left panel:** Violin plots comparing gene expression levels of ERH and MRPS33 between patients with Alzheimer's disease and control across datasets included in the study (male cohort). **Right panel:** scatter plot with linear fit showing Pearson correlation analysis between ERH and MRPS33 across datasets included in the study (male cohort).

**Figure 3:**
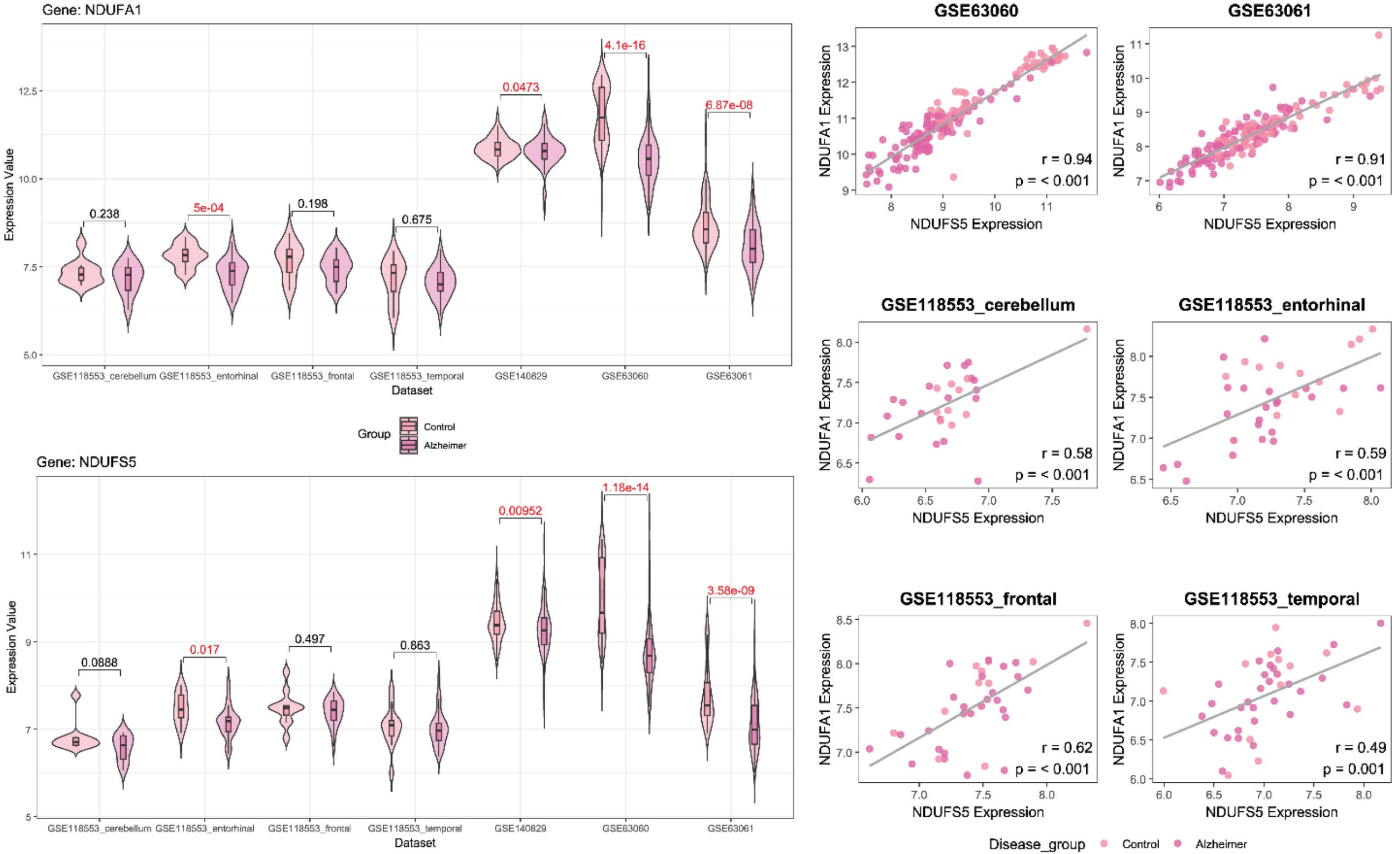
** Left panel:** Violin plots comparing gene expression levels of NDUFA1 and NDUFS5 between patients with Alzheimer's disease and control across datasets included in the study (female cohort). **Right panel:** scatter plot with linear fit showing Pearson correlation analysis between NDUFA1 and NDUFS5 across datasets included in the study (female cohort).

Following feature selection, two genes, ERH and MRPS33, were identified in the male cohort (Figure 2), whereas NDUFA1 and NDUFS5 were screened in the female cohort (Figure 3). ERH and MRPS33 as well as NDUFA1 and NDUFS5 exhibited generally moderate and above levels of correlation across all datasets regardless of tissue type (all P < 0.001).

Both ERH and MRPS33 were downregulated in the AD group compared to the control group. However, they were significantly differentially expressed only in two out of three blood-derived datasets, namely GSE63060 and GSE63061 (all P < 0.001), as well as several brain tissues (GSE118553: entorhinal cortex and frontal cortex; all P < 0.05). In addition, ERH was significantly downregulated in the AD group in the frontal cortex (P = 0.00713) but not in the temporal cortex (P = 0.0749), whereas expression levels of MRPS33 were significantly lower in the AD group in the temporal cortex (P = 0.0227) but not in the frontal one (P = 0.145). Finally, in the female cohort, NDUFA1 and NDUFS5 were downregulated in patients with AD compared to controls. Notably, statistically significant differences were observed in all blood-based gene expression datasets (all P < 0.05) and only in the entorhinal cortex dataset (P = 0.017 and P < 0.001 for NDUFS5 and NDUFA1, respectively).

### Age

Across multiple datasets, individuals with AD were generally older than controls, with several statistically significant differences (Table 2). In GSE140829, females with AD were significantly older compared to controls (73.36 ± 6.68 vs 73.05 ± 7.33 years, P < 0.001), though no difference was observed in males (P = 0.245). In both GSE63060 and GSE63061, significant age differences were observed in females (71.94 ± 6.53 vs 75.02 ± 6.76, P = 0.004, and 74.84 ± 6.21; 81 vs 78.16 ± 7.19, P < 0.001, respectively), while males with AD were significantly older than controls only in the GSE63060 dataset (73.02 ± 6.07 vs 75.80 ± 6.24, P = 0.037). Similar trends were observed in the brain tissue data (GSE118553): males with AD were significantly older compared to controls in the temporal cortex (67.68 ± 16.01 and 81.00 ± 12.00, P = 0.004), entorhinal cortex (74.08 ± 8.98 and 84.21 ± 10.45, P = 0.030), and frontal cortex (70.58 ± 13.15 and 81.07 ± 11.87, P = 0.043). As for females, statistically significant differences in terms of age were observed only in the frontal cortex (67.10 ± 18.43 and 83.96 ± 8.87, P = 0.019). A linear regression analysis was conducted to assess the association between the identified genes and age. In male cohort, no significant associations were observed between ERH or MRPS33 in any dataset except for AD patients in the GSE140829 dataset and GSE118553 (cerebellum) (S Figure 1). In contrast, there were significant associations between age and NDUFA1 expression levels in GSE63060 (both control and AD groups), GSE630361 (control group), and GSE118553 - entorhinal cortex (control group), whereas NDUFS5 was associated with age only in the control group comprising the GSE63060 dataset (S Figure 2).

### Model Development

**Figure 4:**
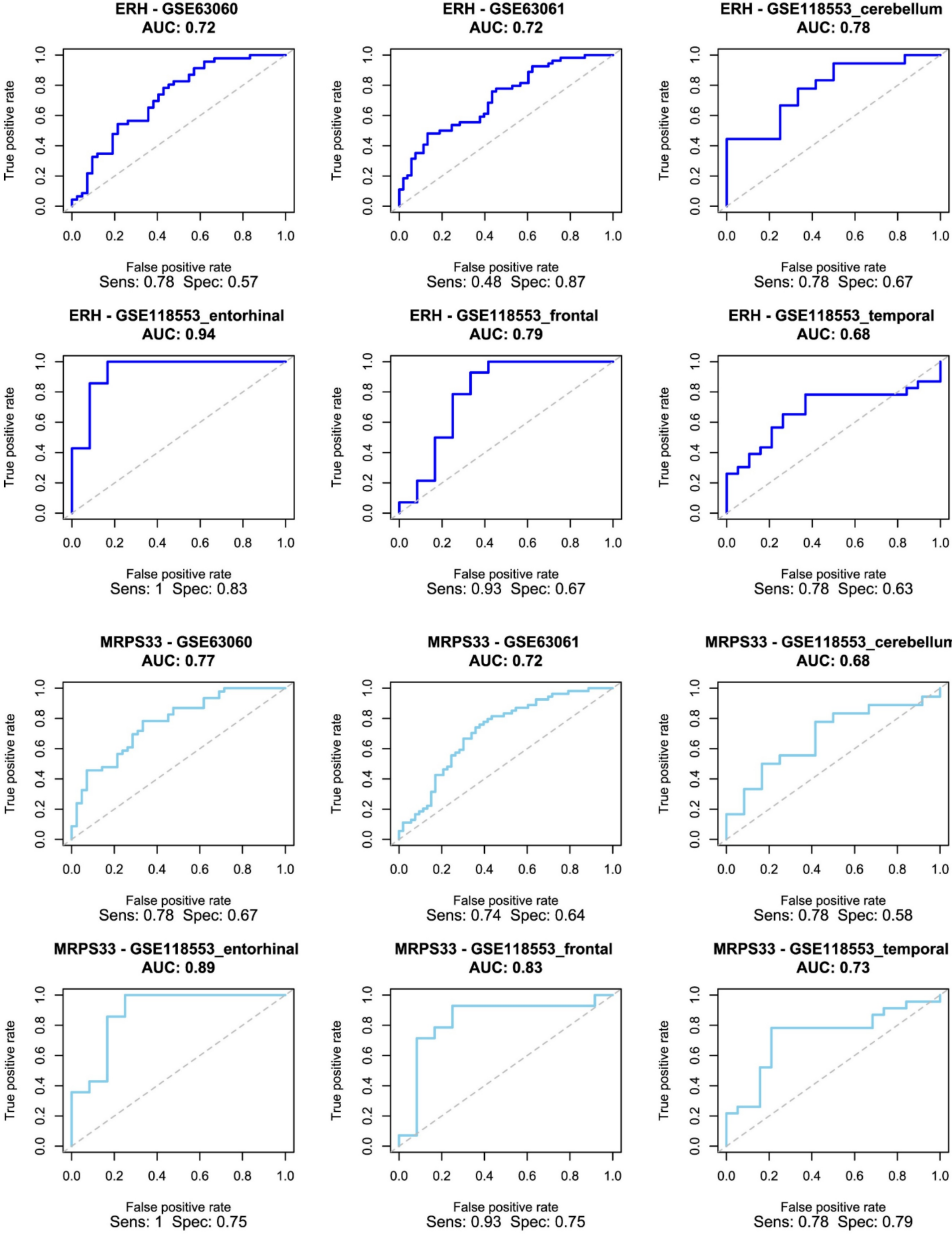
Receiver Operating Characteristic (ROC) curve for logistic regression models trained using ERH (top two rows) and MRPS33 (bottom two rows) in male cohort.

**Figure 5:**
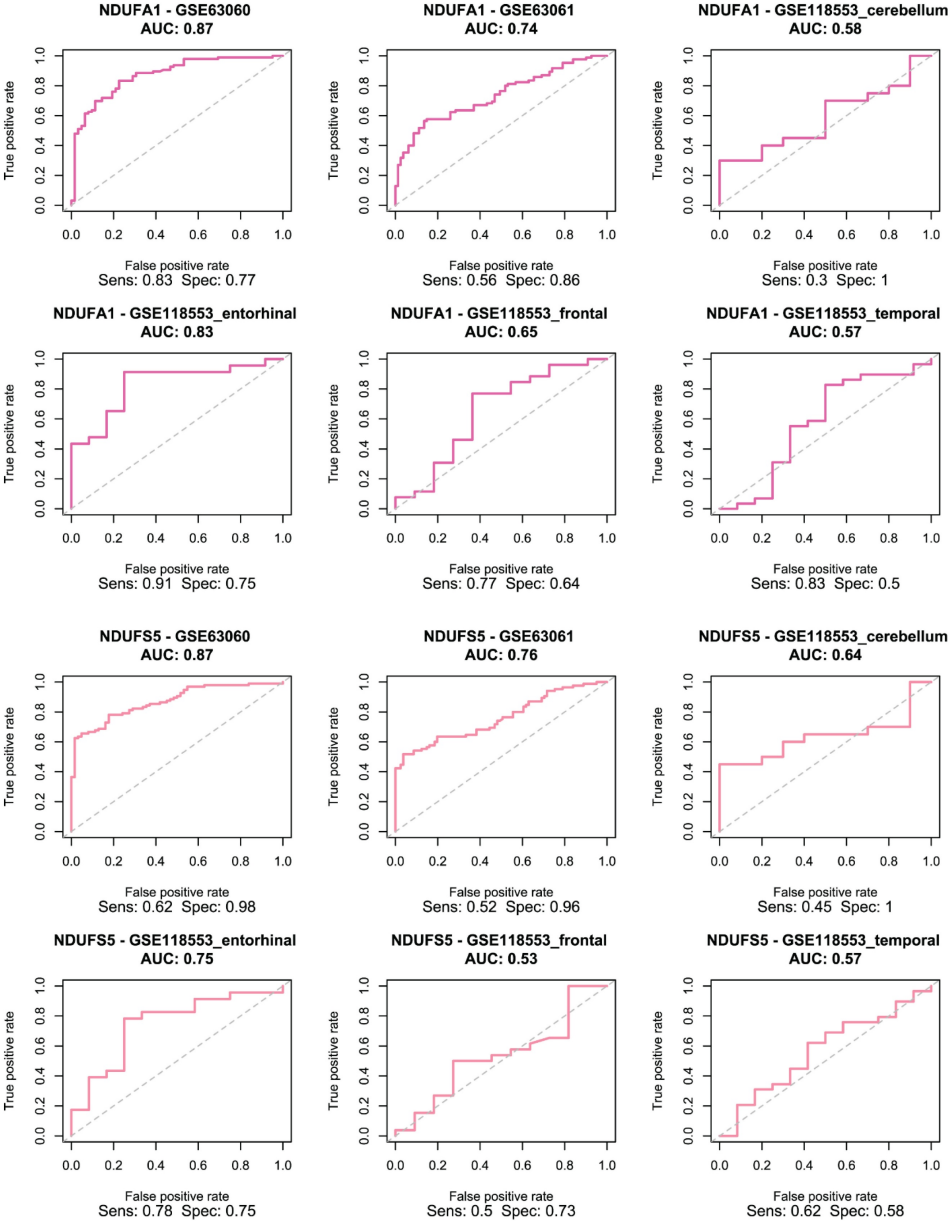
Receiver Operating Characteristic (ROC) curve for logistic regression models trained using NDUFA1 (top two rows) and NDUFS5 (bottom two rows) in female cohort.

The selected genes were used to train and validate a logistic regression model. As demonstrated in Figure 4, ERH and MRPS33 showed good predictive performance across all male-specific datasets, reaching AUC of 0.72 and 0.77 in the first external validation set (GSE63060) and AUC of 0.72 and 0.72 in the second one (GSE63061). Moreover, their predictive performance was even higher in the GSE118553 with both genes having an AUC close to 0.9 in the entorhinal cortex. Notably, performance of models trained on ERH and MRPS33 did not marginally decline when predicting asymptomatic AD (S Figure 3). Specifically, ERH showed optimal sensitivity and specificity in cerebellum (AUC: 0.8) and entorhinal cortex (AUC: 0.77), whereas MRPS33 achieved an AUC of 0.7 in cerebellum, AUC of 0.8 in entorhinal cortex, and AUC of 0.71 in temporal cortex. Both NDUFA1 and NDUFS5 showed great performance in the first validation set and good performance in the second validation set, with AUC of 0.87 and 0.74-0.76, respectively (Figure 5). Moreover, the models were able to reliably predict AD in the entorhinal cortex of the third validation set (AUC of 0.83 for NDUFA1 and 0.75 for NDUFS5). However, their performance declined in the cerebellum, frontal cortex, and temporal cortex. Similarly, when predicting asymptomatic AD, models could not reliably differentiate between cases and controls in almost all brain regions (S Figure 4). Only the model trained using NDUFA1 exhibited a good predictive performance in one of the datasets (entorhinal cortex: AUC of 0.77).

### Experimental Validation

**Figure 6:**
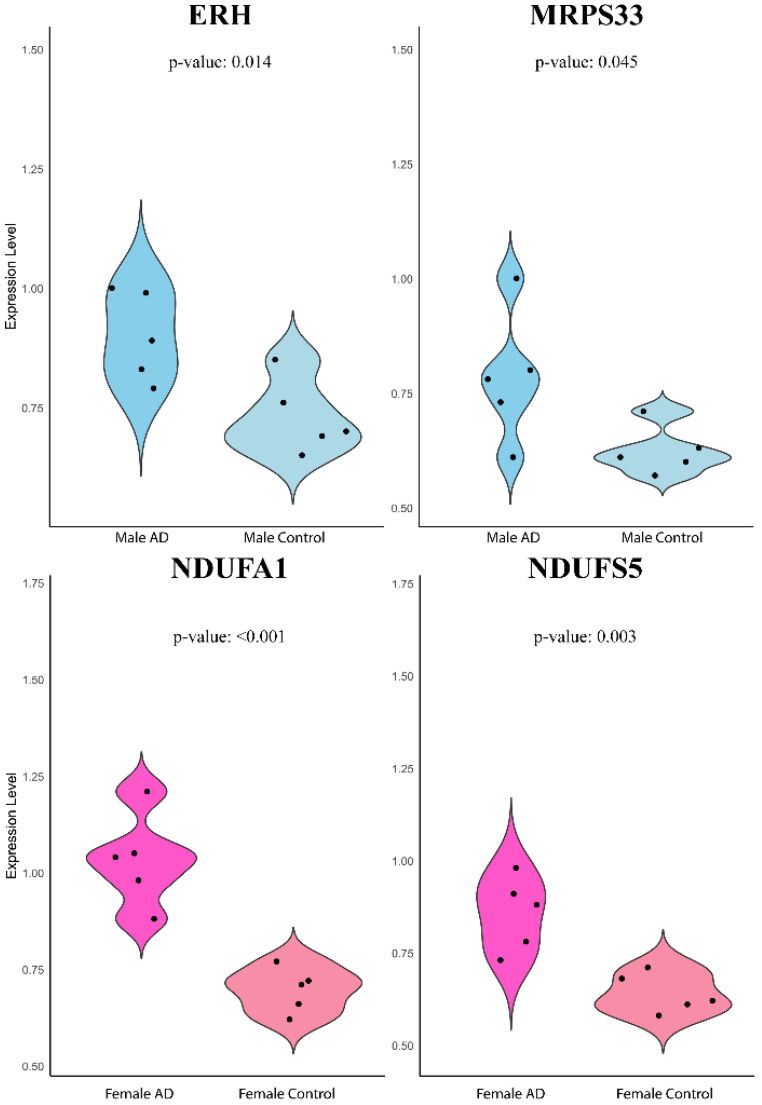
Violin plot of quantitative real-time PCR validation of the expression levels of ERH, MRPS33, NDUFA1, and NDUFS5 relative to GAPDH based on 2-ΔΔCT method.

The expression levels of ERH and MRPS33 in males as well as NDUFA1 and NDUFS5 in females were evaluated in whole venous blood samples of patients with AD and healthy controls without any signs of dementia (Figure 6). Contrary to earlier findings, all four genes were significantly higher in the AD group than in the control group (ERH: P = 0.014, MRPS33: P = 0.045, NDUFA1: P < 0.001, NDUFS5: P = 0.003).

## Discussion

Our study identified ERH & MRPS33 and NDUFA1 & NDUFS5 as biomarkers of AD in males and females, respectively. Performance of the male models trained on ERH and MRPS33 was strong: AUCs of 0.72-0.77 in external blood validations (GSE63060, GSE63061) and nearly 0.90 in the entorhinal cortex of GSE118553. Notably, predictive accuracy remained high for asymptomatic AD in cerebellum (AUCs 0.80 for ERH; 0.70 for MRPS33) and entorhinal cortex (0.77 and 0.80, respectively). Female-specific models (NDUFA1 and NDUFS5) similarly achieved good performance in the first validation set (AUC 0.87) and good performance in the second set (AUC 0.74-0.76), as well as in entorhinal cortex prediction (AUCs 0.83 for NDUFA1; 0.75 for NDUFS5). The four identified genes were downregulated in blood and four brain regions, yet strikingly they were upregulated in our PCR evaluation of peripheral blood.

In studying sex-specific differences in AD, bioinformatics methods are instrumental in processing and interpreting gene expression data 16. First, raw gene expression datasets underwent preprocessing, such as normalization, to ensure data comparability. Differential expression analysis was then conducted to identify genes that varied significantly between AD and normal samples in males and females. By performing a meta-analysis of sex-specific DEGs across three independent blood-derived GEO datasets, we mitigated dataset-specific biases in sample handling, platform variability, and cohort demographics. Unlike merging datasets, which can introduce batch effects, or relying on a single dataset, meta-analysis utilizes the combined statistical power of multiple experiments 17-19. This approach increases generalizability and reproducibility, ensuring that the identified genes (ERH, MRPS33 for males; NDUFA1, NDUFS5 for females) display consistent disease-related expression changes across various cohorts. Importantly, models trained on the identified genes achieved good predictive performance in all blood datasets and at least one brain tissue dataset. Bioinformatics analysis also included pathway enrichment analysis, which can reveal biological pathways influenced by the identified genes. Machine learning complements bioinformatics by analyzing these gene expression profiles to build predictive models. Machine learning algorithms learn patterns from the data to predict disease development 20, 21. We employed 10-fold cross-validated logistic regression model to build sex-specific classifiers based on our selected genes. The linear nature of these models ensures interpretability without relying on complex interpretation tools, such as SHAP and LIME, allowing direct assessment of each gene's contribution to AD risk prediction. This integrated approach enables the discovery of sex-specific biomarkers and molecular pathways associated with AD, advancing our understanding of sex-specific differences in AD progression. The integration of machine learning with sex-specific neurobiological research holds promise for advancing precision medicine in AD and improving diagnostic modalities for individuals based on their unique gene expression characteristics.

ERH is a small, highly conserved nuclear and nucleolar protein that promotes proper mitotic chromosome alignment. However, it has been mostly studied in the field of cancer and its association with neurogenerative diseases is unclear 22, 23. Mitochondrial ribosomal protein 33 (MRPS33) is a nuclear-encoded component of the mitochondrial ribosome, essential for mitochondrial translation and oxidative phosphorylation 24. Disruption of MRPs impairs ribosome assembly and oxidative phosphorylation, linking this gene's expression alterations to neurodegenerative diseases. It is worth mentioning that the identified DEGs in both female and male cohorts were involved in ribosome pathways. Ribosomal dysfunction impairs mitochondrial translation, disrupts oxidative phosphorylation, and contributes to neuronal energy failure and amyloidogenic stress in AD 25, 26. NDUFA1 and NDUFS5 are nuclear-encoded subunits of mitochondrial complex I and belong to the NADH-ubiquinone oxidoreductase family. Proteomic studies consistently report the downregulation of NDUFA1 and NDUFS5 in early-onset and late-onset AD, reflecting destabilization of complex I 27. Machine-learning-based analyses in blood cohorts identified both NDUFA1 and NDUFS5 as robust diagnostic biomarkers across sexes 28, and their combined inclusion with age yielded AUCs >0.72 for late-onset AD prediction 29.

Given the known risks of aging in Alzheimer's disease, we evaluated correlations between age and transcript levels. In our male cohort, ERH and MRPS33 showed no significant age associations as except within the AD subgroup of GSE140829 and the cerebellum dataset of GSE118553. In females, however, NDUFA1 expression inversely correlated with age in multiple datasets (GSE63060, GSE63061 control, and GSE118553 entorhinal cortex), and NDUFS5 was associated with age in the GSE63060 control group. These findings may indicate that, particularly for complex I subunits, aging may modulate expression in healthy individuals, suggesting the need to adjust for age in future biomarker evaluations.

Across analyzed datasets, ERH and MRPS33 as well as NDUFA1 and NDUFS5 were significantly downregulated in male and female AD patients compared to controls, respectively. However, the opposite trend was observed in our PCR validation. This is likely attributed to differences in cohorts and/or sample size. It is difficult to establish the exact reason since the original studies did not provide any information on treatment, comorbidities, etc. First, given the location of our institution, only Chinese patients with AD and controls were included in the study. Second, such large discrepancies may be due to existing comorbidities, received treatment or disease stage. Third, sample size is smaller compared to that of the included datasets and had only 10 cases and 10 controls, which were further divided based on sex. The identified genes were associated with good predictive performance in most of the included datasets, namely blood datasets and entorhinal cortex. Performance decline in the other regions as well as during asymptomatic AD shows regional and stage-specific limitations of constructed models. The strong performance of all predictive models in the entorhinal cortex can likely be attributed to the region's central and early involvement in Alzheimer's disease pathogenesis. As consistently shown across numerous studies, the entorhinal cortex is among the first brain regions to undergo structural, cellular, and molecular changes, including Tau accumulation and layer II neuronal degeneration, even during the preclinical stage of AD 30-32. Therefore, gene expression patterns in the entorhinal cortex may offer a clearer “picture” of AD-related molecular processes, which may explain why our models demonstrated superior and consistent predictive performance in this region.

Given the number of included number of samples, datasets, tissue specimens and good performance of constructed models based on the identified genes, we demonstrate that the identified genes are robust and generalizable biomarkers for AD. However, our study is not without limitations. First, all PCR validations were conducted in a single prospective cohort without accounting for covariates. Second, datasets we used did not report medication use, comorbidities, disease stage or lifestyle factors, and thus these variables could not be adjusted for. Third, the number of samples in the gene expression datasets or in our cohort for PCR validation was modest, which could potentially limit the reliability of our findings. Finally, while our logistic models performed well in various external validation datasets, prospective validation in independent clinical cohorts is essential.

## Conclusion

Through meta-analysis of multiple datasets, we identified ERH and MRPS33 as well as NDUFA1 and NDUFS5 as robust biomarkers for sex-specific prediction models for AD. Logistic regression models based on these genes demonstrated good prediction performance in both peripheral blood datasets and in various brain regions, particularly the entorhinal cortex. However, qPCR analysis yielded opposite results of gene expression levels in AD and control groups.

## Supplementary Material

Supplementary figures.

## Figures and Tables

**Table 1:** List of primers

Primer name	Reference sequence	Nucleotide sequence (5'-3')		Tm Value	GC Content%	Length (bp)
GAPDH	NM_002046	sense	CATCATCCCTGCCTCTACTGG	59.4	57.1	259
		antisense	GTGGGTGTCGCTGTTGAAGTC	60.1	57.1	
NDUFA1	NM_004541	sense	GTGCTTGTTGATTCCAGGACTG	59.6	50	104
		antisense	CCATCAGACTCCAGTGATACCC	58.3	54.5	
NDUFS5	NM_004552	sense	TAACATAGATCGATGGTTGACAATC	57.1	36	170
		antisense	GAAGCAAACACTCTACGAAATCATC	58.8	40	
ERH	NM_004450	sense	TATGCTGACTACGAATCTGTGAATG	59	40	225
		antisense	CACGTAGATCTTCTCTTTAATCCAG	57	40	
MRPS33	NM_016071	sense	GGTGAAGTCACCAGGCCTACTA	61.2	54.5	130

**Table 2:** Age of females and males in each dataset.

Dataset	Specimen	Gender	Control Group Age (Mean ± SD; n)	Alzheimer's Disease Group Age (Mean ± SD; n)	P value
GSE140829	Blood	Male	74.05 ± 5.67; 110	72.94 ± 6.87; 100	0.245
		Female	73.36 ± 6.68; 139	73.05 ± 7.33; 104	<0.001
GSE63060	Blood	Male	73.02 ± 6.07; 42	75.80 ± 6.24; 46	0.037
		Female	71.94 ± 6.53; 62	75.02 ± 6.76; 96	0.004
GSE63061	Blood	Male	75.98 ± 5.72; 53	77.46 ± 5.79; 54	0.186
		Female	74.84 ± 6.21; 81	78.16 ± 7.19; 85	<0.001
GSE118553	Temporal Cortex	Male	67.68 ± 16.01; 19	81.00 ± 12.00; 23	0.004
		Female	71.91 ± 18.68; 12	83.55 ± 9.69; 29	0.118
	Cerebellum	Male	69.17 ± 12.28; 12	79.40 ± 10.90; 18	0.057
		Female	73.10 ± 18.28; 10	85.20 ± 9.34; 20	0.074
	Entorhinal Cortex	Male	74.08 ± 8.98; 12	84.21 ± 10.45; 14	0.030
		Female	71.50 ± 21.30; 12	83.90 ± 9.59; 23	0.216
	Frontal Cortex	Male	70.58 ± 13.15; 12	81.07 ± 11.87; 14	0.043
		Female	67.10 ± 18.43; 11	83.96 ± 8.87; 26	0.019
Present study	Blood	Male	75.4 ± 7.06; 5	72.4 ± 6.11; 5	0.493
		Female	72.4 ± 3.91; 5	71.0 ± 4.12; 5	0.832

## Data Availability

Detailed information about the included patients and code can be obtained from the author upon reasonable request.
