# Supplementary material for: Sex-Specific Prediction Models of Alzheimer's Disease: A Gene Expression Analysis
Source: Int J Med Sci. 2026 Jan 30;23(3):852–63. doi: 10.7150/ijms.122666 (PMC12964574; doi:10.7150/ijms.122666)

Supplementary Figure 1 Linear regression analysis between MRPS33/ERH and age in male cohort.

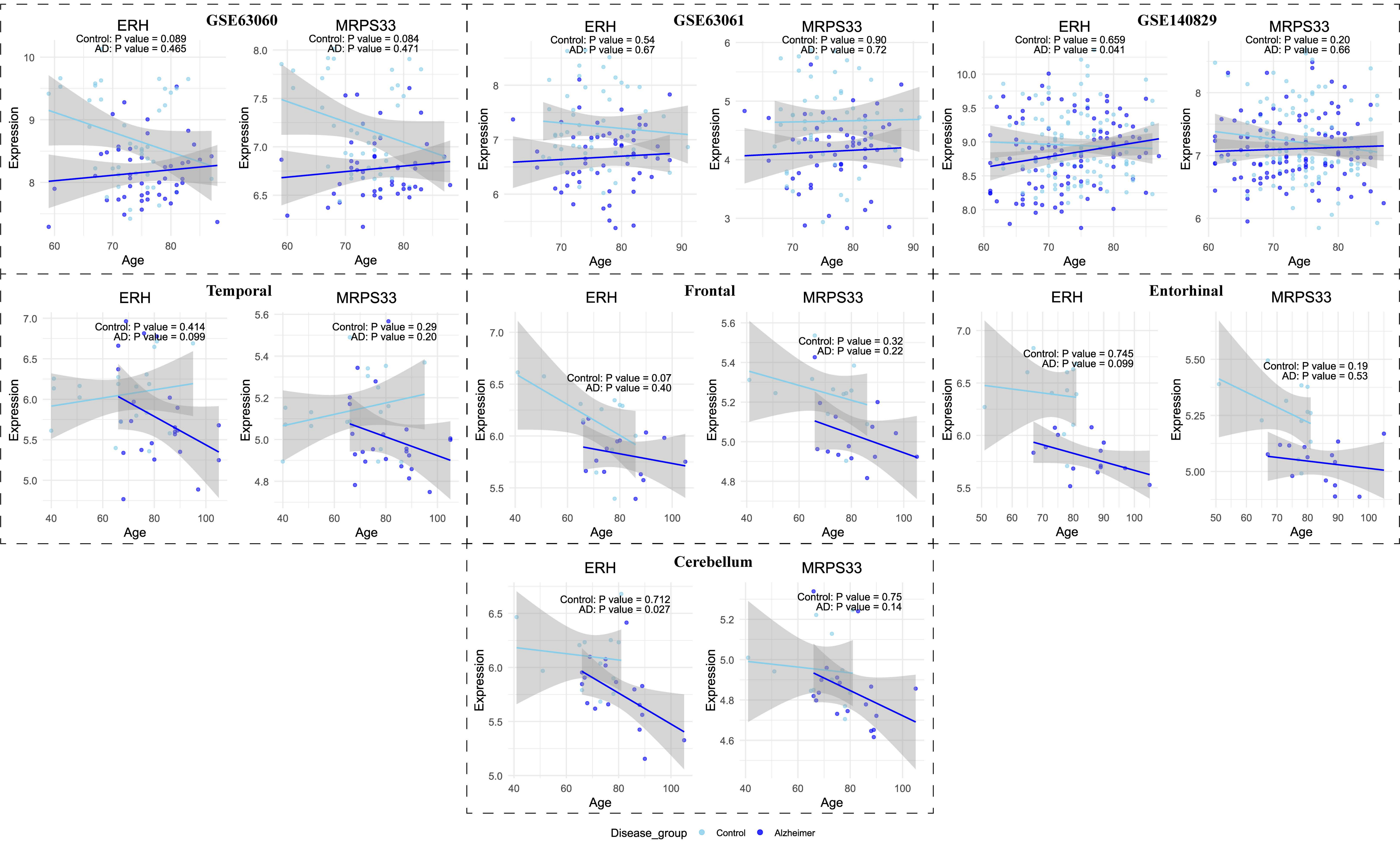

Supplementary Figure 2 Linear regression analysis between NDUFA1/NDUFS5 and age in female cohort.

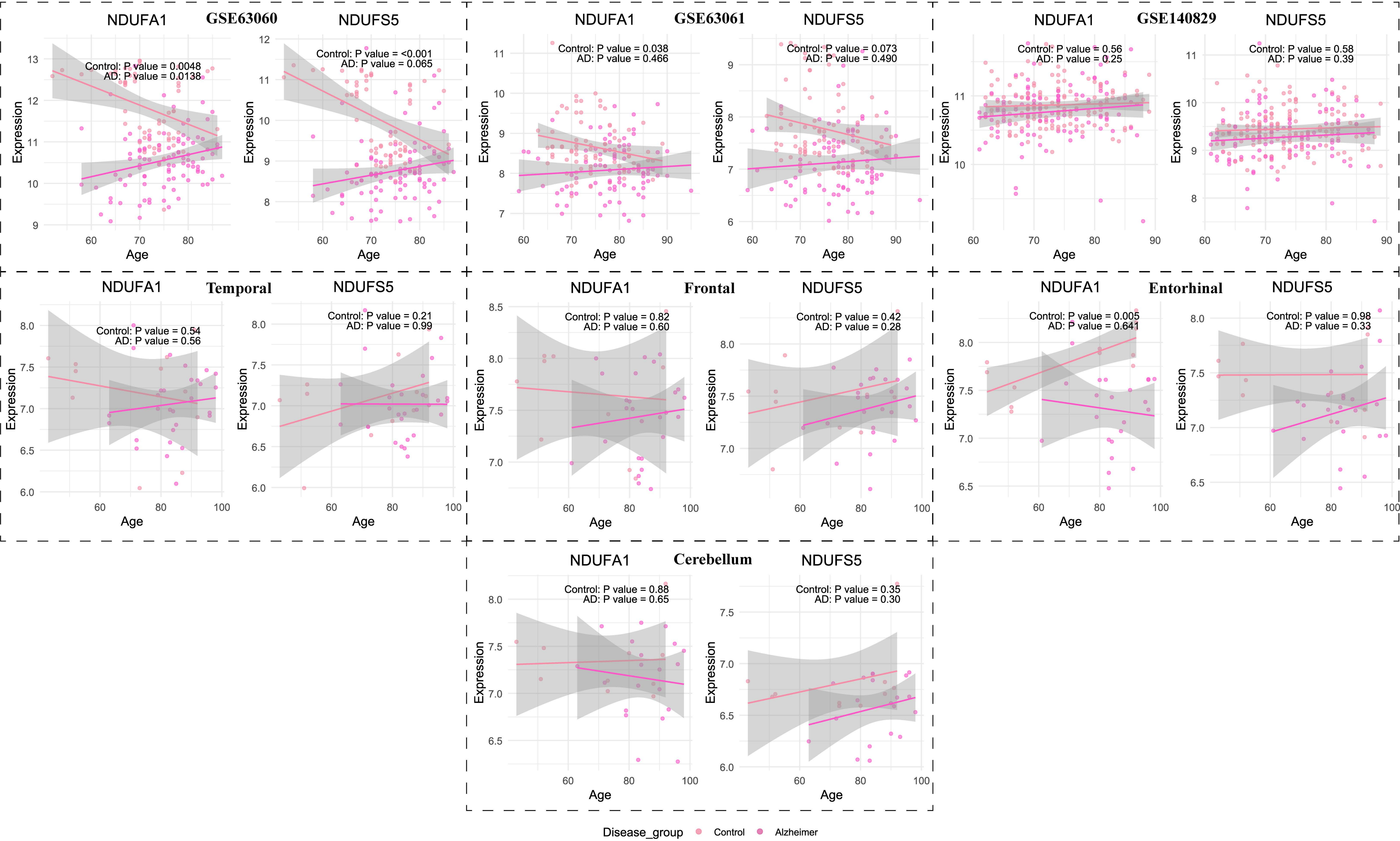

Supplementary Figure 3 Receiver Operating Characteristic (ROC) curve for logistic regression models trained using ERH (top two rows) and MRPS33 (bottom two rows) in male cohort (brain tissue, asymptomatic patients with Alzheimer's disease).

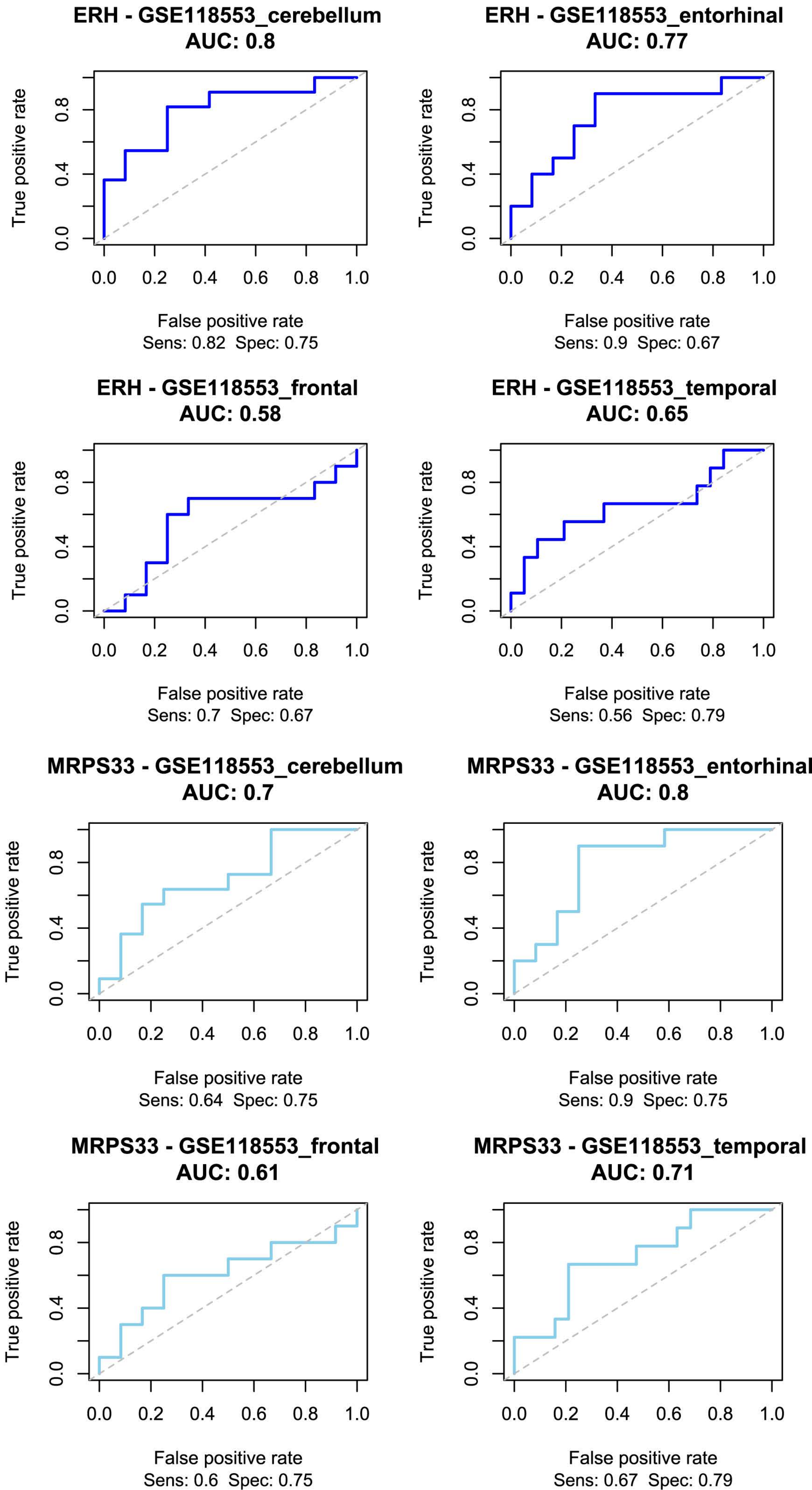

Supplementary Figure 4 Receiver Operating Characteristic (ROC) curve for logistic regression models trained using NDUFA1 (top two rows) and NDUFS5 (bottom two rows) in female cohort (brain tissue, asymptomatic patients with Alzheimer's disease).

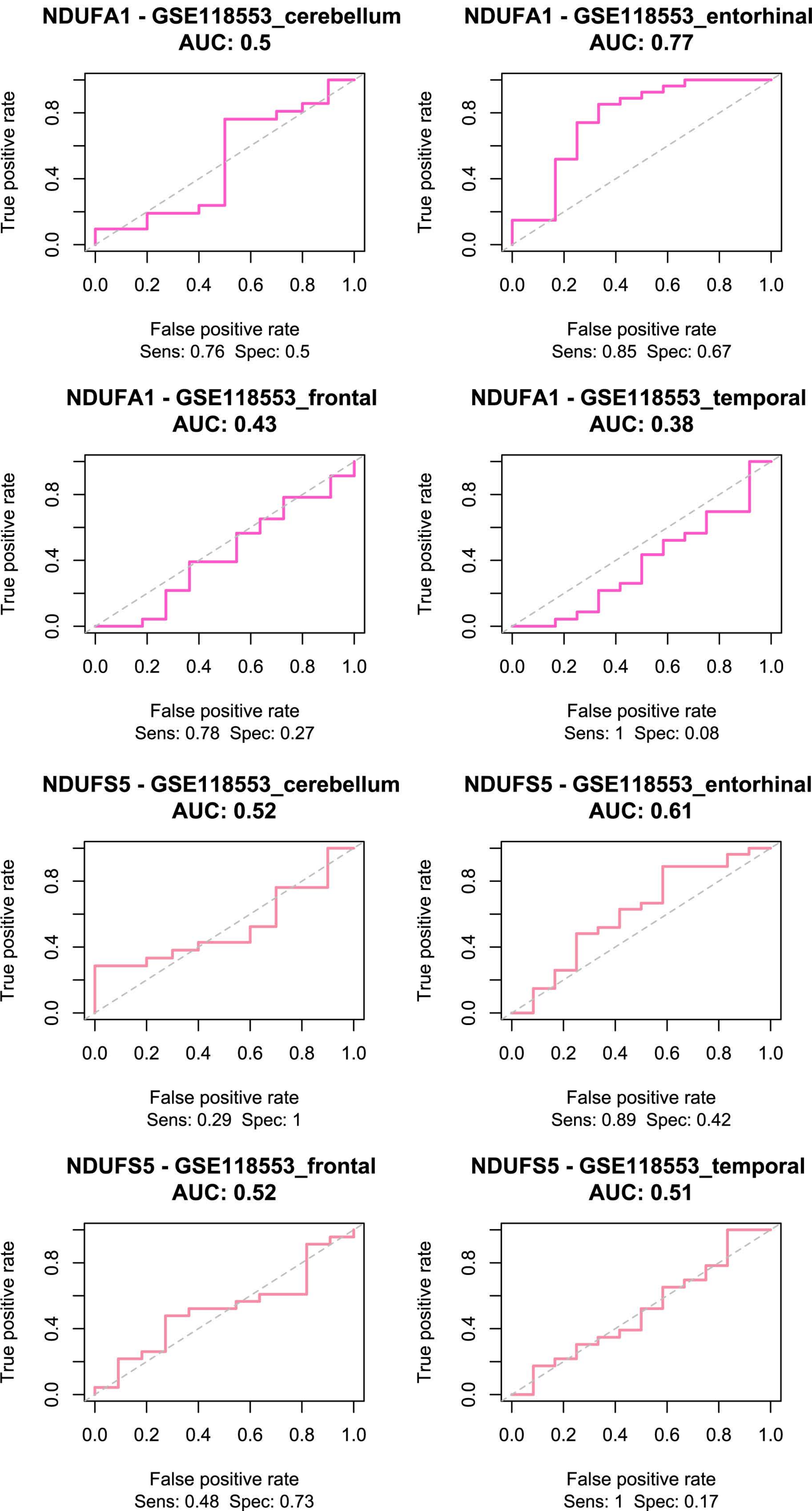

Supplement: Supplementary file 1 — Supplementary figures. [file ijmsv23p0852s1.pdf]
